# Magnetic nanofibers based bandage for skin cancer treatment: a non‐invasive hyperthermia therapy

**DOI:** 10.1002/cnr2.1281

**Published:** 2020-09-03

**Authors:** Kaushik Suneet, Tamasa De, Annapoorni Rangarajan, Shilpee Jain

**Affiliations:** ^1^ Centre for Biosystems Science and Engineering Indian Institute of Science Bangalore Karnataka India; ^2^ Department of Molecular Reproduction, Development and Genetics Indian Institute of Science Bangalore Karnataka India

**Keywords:** drug delivery, drug‐resistant cells, hyperthermia, magnetic nanofibers, skin cancer

## Abstract

**Background:**

The treatment of non‐melanoma skin cancer and deadliest malignant melanoma skin cancer are the fifth and ninth most expensive treatments in Medicare, respectively. Moreover, the recurrence of cancer after currently available therapies, that is, surgery or radiotherapy, reduces the patient's life expectancy.

**Aims:**

In view of this, we fabricated magnetic nanofibrous mat‐based bandage to treat skin cancer non‐invasively using an external alternating current (AC) magnetic field induced hyperthermia.

**Methods:**

The Fe_3_O_4_ nanoparticles incorporated polycaprolactone (PCL) fibers based bandages were fabricated using the electrospinning technique. The efficacy of the bandage was investigated in vitro using parental/doxorubicin hydrochloride (Dox)‐resistant HeLa cells and in vivo using BALB/c mouse model in the presence of an external AC magnetic field (AMF).

**Results:**

The PCL‐Fe_3_O_4_ fibrous mat‐based bandages dissipate heat energy locally on the application of an external AMF and increase the surrounding temperature in a controlled way up to 45°C in a few mins. The in vitro study confirms the elevated temperature could kill parental and Dox‐resistant HeLa cells significantly. As the activity of Dox enhanced at a higher temperatures, more than 85% of parental HeLa cells were dead when cells incubated with Dox contained fibrous mat in the presence of AMF for 10 minutes. Further, we confirm the full recovery of chemically induced skin tumors on BALB/c mice within a month after five hyperthermic doses for 15 minutes. Also, there was no sign of inflammation and recurrence of cancer post‐therapy.

**Conclusion:**

The present study confirms the PCL‐Fe_3_O_4_ nanofibrous based bandages are unique and compelling to treat skin cancer.

## 1 INTRODUCTION

The severity of the cancer is always defined as a number of deaths per year. However, in the case of skin cancer, the number of deaths is enormously less than the number of cases each year. Still, the expensive treatment of skin cancer puts a considerable burden on Medicare.^1^, ^2^ Being the largest organ of the body, the chances of getting excessive exposure to ultra‐violet (UV) or sun are unavoidable, which results in a large number of skin cancer cases per year.^3^, ^4^, ^5^ Skin cancers are categorized in malignant melanoma (MM) and non‐melanoma skin cancer (NMSC). MM is the deadliest cancer with a higher rate of metastasis.^6^ NMSC is the most common cancer, which includes basal cell carcinoma (BCC) and squamous cell carcinoma (SCC) skin cancers. Besides sun or UV exposure, organ transplants or immunocompromised patients are found to be more susceptible to NMSC.

There are several therapies to treat skin cancers. Surgery is the primary option for skin cancer treatment, in which the infected skin lesion is removed with precision. In the advanced Mohs micrographic surgery (MMS), thin layers of skin cancer precisely excise and analyze under the microscope with color‐coded margins by utilizing tangentially cut frozen‐section histology.^7^ Nevertheless, cosmetic defect or accompanying disease,^8^ recurrence of cancer,^9^ the opening of the wound,^10^ wound infection,^11^ and the requirement of graft for wound closure are the major challenges of the surgery.^12^ If surgical excision is not possible due to the patient's medical condition or hard to operate the site, for example, the eyelids, inner and upper canthi, the ear, and nose, radiation therapy is considered for the treatment.^13^, ^14^ In radiation therapy, high energy radiations, such as X‐rays and proton rays, are used to kill cancer‐spreading cells. However, there is some safe limit of radiation doses one can have in their entire lifetime; therefore, a person cannot receive radiation therapy if that person already has taken maximum doses.^15^ Radiation therapy can also increase the risk of subsequent skin cancer post‐therapy.^5^, ^16^ Furthermore, topical chemotherapy involves the use of chemodrug locally to kill cancerous cells, which can be used to treat only NMSC. The major drawback of this therapy is that after a few doses, cancerous cells block chemodrugs induced apoptosis and become drug resistant.^17^ This tendency of the cells makes the treatment difficult to kill tumor cells using chemodrugs further. The photodynamic therapy (PDT) involves the activation of a photosensitizing agent by light ranging from UVA to near‐infrared wavelength.^18^ The photosensitizer reaches an excited state that undergoes a reaction with ambient oxygen to create reactive oxygen species (ROS). These ROS then react with intracellular components and induce cell inactivation, followed by cell death. Since this therapy requires photosensitizing drug and light sources, consequently, it cannot be used for disseminated cancer.^19^ Also, poor penetration of light in the case of melanoma cancer is the major limitation of this therapy. Another promising approach to treat cancer is hyperthermia therapy, which includes the delivery of heat energy to increase the surrounding temperature (40°C‐45°C). It is reported that the hyperthermia induces endoplasmic reticulum‐mediated apoptosis in melanoma and NMSC cells.^20^ Further, hyperthermia can reverse the chemodrug resistance of the cells and enhance the drug activity.^21^ The low doses of hyperthermia can enhance the anticancer drug efficacy by modifying responsible gene expression, for example, reducing the MDR1 gene expression, which increases drug sensitivity.^22^ Therefore, the combination of hyperthermia and drug could enhance the efficacy of the treatment by reducing the cellular resistivity toward the anticancer drug.

The continuing advancement in technology provides ways to design smart systems to deliver heat to a localized area using radiofrequency radiation, laser ablation, microwaves, ultrasound waves, and magnetic field. The magnetic hyperthermia (MH) is based on the principle of delivering heat energy locally using magnetic materials with the application of an external alternating magnetic field (AMF).^23^, ^24^, ^25^ The hyperthermia efficiency is dependent on the type of magnetic material, shape, size, and magnetic field strength. It is reported that the heating capabilities of a one‐dimensional (rod shape) material is better than its zero‐dimensional shapes (spherical particle).^26^ Motoyama et al investigated the dependence of heating ability on the particle size and the AMF strength.^27^ They found that the particles >1000 nm size with smaller specific‐surface‐area show a strong correlation of the heating capacity with the intensity of the AMF power (100‐300 kHz and 2‐32 kA/m) compared to particles ~100 nm size, which show weaker correlation. To date broad range of iron oxide‐based magnetic nanoparticles (MNPs) have been reported in the literature to achieve localized heating, for example, γ‐Fe_2_O_3_, γ‐MFe_2_O_3_ (M = Co, Ni, Mn), Fe_3_O_4_, and graphene‐Fe_3_O_4_ composite.^28^, ^29^, ^30^, ^31^

Several groups reported the efficacy of MNPs based systems using various models in vitro and in vivo. For instance, Fantechi et al studied the efficacy of cobalt‐doped ferrite (Co‐doped HFt) nanoparticles using the B6 melanoma cell line as a model in vitro.^32^ They reported apoptotic cellular death when cells were exposed to magnetic field of 17.0 kA/m and 183 kHz with Co‐doped HFt for 30 minutes. Despite various studies of MNPs for hyperthermia therapy, there is no substantial evidence of the use of such systems clinically for the treatment of cancer due to their physical limitations; that is, agglomeration, non‐uniform heating, and toxicity related to high dose or long term accumulation in the body.^33^, ^34^, ^35^ The higher surface energy associated with MNPs leads to frequent agglomeration and the uneven distribution of agglomerated MNPs gives rise to non‐uniform heating at the site of treatment. Additionally, the long‐term accumulation of MNPs in the body induces toxicity, which could lead to an adverse side effect of the therapy. Herein, we are proposing magnetic nanofibrous based bandage to treat skin cancer using hyperthermia. The magnetic nanofibrous based bandages overcome the challenges associated with suspended MNPs and provide effective treatment for skin cancer in a short period.^36^, ^37^ Additionally, the localized heating reduces the associated side effects of the therapy and can be used easily in the case of multiple tumors.

## 2 MATERIALS AND METHODS

### 2.1 Materials

Polycaprolactone (PCL, number average molecular weight *M*_n_ = 80 000), Fe_3_O_4_ nanoparticles (spherical 50‐100 nm diameter), fetal bovine serum (FBS), 0.25% trypsin‐ethylenediaminetetraacetic acid (EDTA) solution, 3(4,5‐dimethylthiazol‐2‐yl)‐2,5‐diphenyl tetrazolium bromide (MTT), doxorubicin hydrochloride (Dox), antibiotic‐antimycotic solution, 7,12‐dimethylbenz(a)anthracene (DMBA), and phorbol 12‐myristate 13‐acetate (PMA) were obtained from Sigma‐Aldrich, St. Louis, MI, USA. 2,2,2‐Trifluoroethanol (TFE) was obtained from Spectrochem chemicals, Mumbai, India. Formaldehyde and dimethyl sulfoxide (DMSO) were bought from Fischer Scientific, USA. Dulbecco's modified eagle's medium (DMEM) was purchased from HiMedia, France.

### 2.2 Fabrication of magnetic nanofibrous mat

The PCL and Fe_3_O_4_ MNPs based fibers were fabricated using the electrospinning technique (E spin: Physics Equipment, Chennai, Tamil Nadu, India) as discussed previously.^23^, ^27^ Briefly, MNPs (3%, wt/vol) were mixed in PCL (15%, wt/vol), TFE, and DMSO solution using ultrasonicator before spinning. The volume ratio of TFE to DMSO was kept at 90:10. The distance between the syringe tip and the collector was 7 cm, and 20 kV voltage was applied during the electrospinning. The morphology of the fibrous mat was analyzed using field emission scanning electron microscopy (FESEM). The SEM images were used to determine the diameter distribution of fibers through imageJ software. The hysteresis loss from the PCL‐Fe_3_O_4_ fibrous mat was recorded using a vibrating sample magnetometer (VSM: Lake Shore instrument, Westerville, OH, USA). Further, in the case of drug contained fibers, Dox was added in the PCL solution before electrospinning followed by ultrasonication.^37^ The mats were suspended in phosphate buffer saline (PBS) and placed in the center of the induction coil (Ambrell Easy Heat solution) to measure the change in temperature. An AMF of 3.6 kA/m and frequency 236 kHz were applied for 10 minutes, and the change in temperature was measured using an alcohol thermometer. Further, the fibrous bandage was prepared using surgical tape and each bandage contained a 10 mg fibrous mat.

### 2.3 Cell Culture

HeLa cells were obtained from American Type Culture Collection (ATCC), and the authenticity of the cells was confirmed using short tandem repeat (STR) analysis. A Dox‐resistant variant of cervical cancer HeLa cell line was generated by stepwise selection with doxorubicin as mentioned above.^38^ Briefly, HeLa cells were initially treated with 5 nM Dox. These treated cells were allowed to recover in the absence of Dox and resume proliferation after 1‐week of treatment. A stable Dox‐resistant HeLa cells were finally derived after 6 months of constant culturing in DMEM supplemented with 10% FBS and 1% antibiotic and antimycotic solution and maintained at 37°C with 5% CO_2_ atmosphere with 90% humidity in an incubator (New Brunswick Galaxy 170S). It has been shown earlier that these cells are able to tolerate 2 μM Dox. The cells were trypsinized using 0.25% trypsin‐EDTA solution, and 5 × 10^4^ cells per well were seeded on the gelatine coated glass coverslips. After 24 hours of seeding, the coverslips were transferred carefully in the flat bottom culture tubes followed by the addition of small pieces of 10 mg magnetic fibrous mat before the application of AMF. The parental Hela and Dox‐resistant HeLa cells were divided into four groups. The first group of cells incubated with only fibrous mat (F), second group incubated with fibrous mat containing Dox (F + D), third group incubated with fibrous mat and AMF (F + H), and fourth group incubated with Dox contained fibrous mat and AMF (F + H + D). After treating cells for 10 minutes, cells were incubated with MTT solution (0.5 mg/mL) for about 2 hours. Lastly, the insoluble formed formazan crystals were dissolved in 500 μL DMSO and the optical density was obtained at 570 nm using a plate reader (Tecan Infinite M 1000 Pro).

### 2.4 Skin tumor induction and treatment on BALB/c mice

BALB/c mice (female: 6‐8 weeks old, 20‐22 g average weight) were procured and housed in Central Animal Facility (CAF), Indian Institute of Science (IISc), Bangalore, India. Animal protocols were approved by the Institutional Animal Ethics Committee (IAEC), and animals were maintained according to CPSEA guidelines (a light/dark cycle of 12 hours, temperature 22°C ± 2°C). Mice were provided with food and water *ad libitum*. The skin tumor on BALB/c mice was induced by two‐stage chemical carcinogenesis using DMBA solution in acetone (0.1%, wt/vol) and PMA as a promotor as mentioned in Reference.^10^ The mice were divided into four groups; control group (C group: 4 mice): without induction of tumor, untreated group (UT group: 4 mice): tumor without treatment, only AMF treated group (TH group: 4 mice): application of AMF without bandage, and treated group (TBH group: 12 mice): treated mice with bandage and AMF. The dorsal skin of all the mice was shaved using surgical clippers by gently restraining mice by the tail for no longer than 1 to 2 minutes. One week after hair removal, 100 μL of DMBA (400 nmol) was applied to the shaved area, followed by restraining the mouse for an additional 5 to 10 seconds to allow the acetone solution to evaporate. Control mice received 0.2 mL acetone only. Mice were isolated in disposable biohazard caging for a week following the application of DMBA. After 1 week, 100‐μL PMA was applied twice weekly to the DMBA‐treated area. For around 90 days, all the mice's body weight was recorded at regular intervals to ensure that test and control mice maintain approximately equal rates of weight gain. The treatment was started with these BALB/c mice only when the palpable tumors attained a diameter of 5 mm or higher (as measured by vernier caliper) and those which persisted for 2 weeks or longer. The hair was removed carefully near the tumor before the onset of the treatment. The tumor lesion of all the TBH group mice was appropriately covered using a bandage and subjected to AMF to achieve a temperature of 45°C, whereas the mice of the TH group exposed to AMF without any bandage. The bandage temperature was maintained at 45°C for 15 minutes. This treatment was given to the tumor‐induced mice for three consecutive days, followed by repetition on alternative days for two more sessions, that is, fifth and seventh day. After completion of the treatment, blood samples were collected to check on the liver and kidney functions. After 30 days of the treatment, the mice were sacrificed, and the skin samples were collected and analyzed by performing an hematoxylin and eosin (H&E) stain.

### 2.5 Statistical analysis

All the experimental data obtained using the MTT assay and blood tests are expressed as mean ± standard deviation (M ± SD) and were analyzed by one‐way analysis of variance (ANOVA; SPSS 16.0) for the calculation of significance level of the experimental data (n = 4). The differences were considered statistically significant at *P* ≤ .05.

## 3 RESULTS

### 3.1 Magnetic nanofibrous mat‐based bandage

**FIGURE 1:**
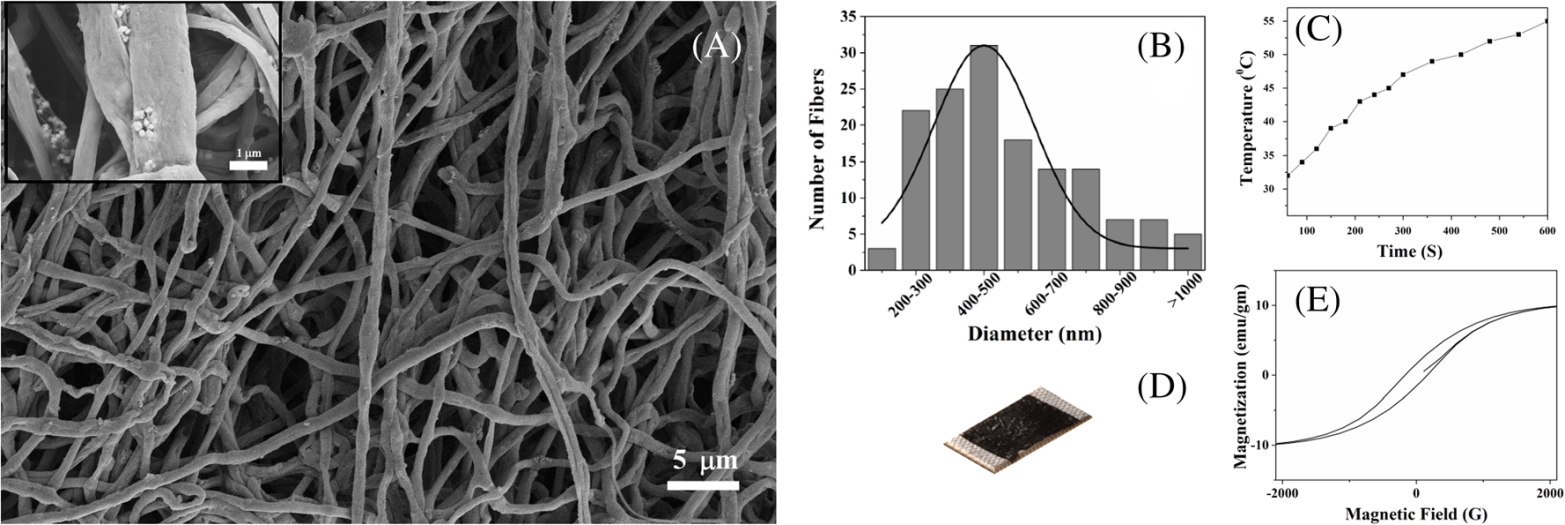
(A) Scanning electron micrographic images of PCL–Fe_3_O_4_ fibrous mat (inset: higher magnification image). (B) The diameter distribution of fibers. (C) The change in temperature of the suspended fibrous mat in 1 × PBS with the application of AMF. (D) Photograph of bandage. (E) Hysteresis curve of fibrous mat. AMF, alternating magnetic field; PCL, polycaprolactone

The magnetic fibrous mat was fabricated using the electrospinning technique. The MNPs were suspended in the PCL‐TFE‐DMSO solution and electrospun on an aluminum foil with 20 kV external electric voltage. The PCL‐Fe_3_O_4_ fibers were continuous, and the MNPs were decorated on the surface of the fibers (Figure 1A). The diameter of fibers varies between 100 and 1000 nm, and its distribution can be described by normal Gaussian distribution, as shown in Figure 1B. The thickness of the deposited fibrous mat on the aluminum foil was controlled by deposition time and the flow rate of the solution. It is evident from the Figure 1C, the 10 mg fibrous mat suspended in 1 × PBS increased surrounding temperature by ~22°C in 10 minutes in the presence of AMF (3.6 kA/m and frequency 236 kHz). Therefore, the 10 mg of the fibrous mat was pasted on the medical grade surgical tape in order to form a bandage, as shown in Figure 1D. Further, MH hysteresis loops infer magnetic nature of the system with the saturation magnetization (MS, emu/g) value 11 emu/g (Figure 1E).

### 3.2 Effect of hyperthermia on Dox‐resistant HeLa cells

**FIGURE 2:**
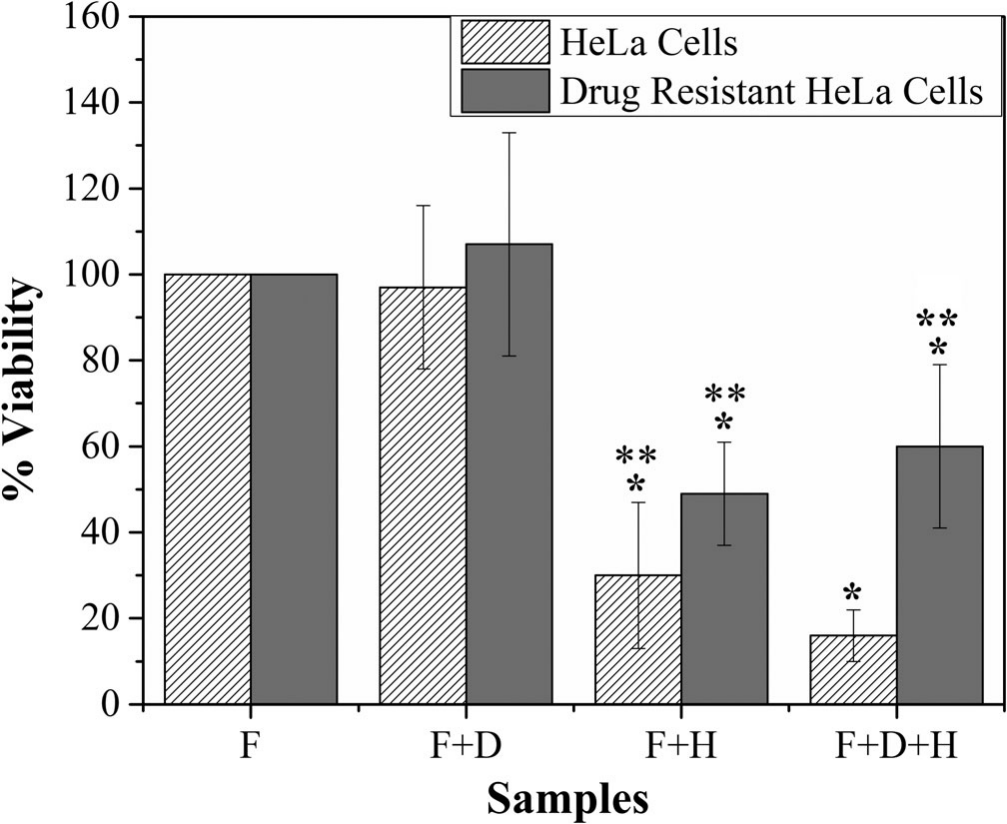
Percent viability of HeLa cells and Dox‐resistant HeLa cells after treatment. * shows the significant difference with respect to control and ** shows the significant difference with respect to HeLa cells incubated with Dox contained fibers and heat (F + D + H) at *P* ≤ .05. F, fibrous mat; D, Dox; H, hyperthermia

The Dox‐resistant HeLa cells and parental HeLa cells were exposed to heat with or without Dox contained fibers using external AMF for 10 minutes duration. The MTT assay was performed to confirm the viability of cells after incubation with heat and Dox. To validate the individual and combined effect of heat and Dox on Dox‐resistant HeLa and parental HeLa cells, the cells divided into four groups. The cells in all the groups incubated with fibrous mat (F), the Dox contained fibrous mat (F + D), heat (F + H), or heat and Dox (F + H + D) for 10 minutes. The viability of the first group of Dox‐resistant HeLa and parental HeLa cells incubated with only fibrous mat (F) was not significantly different (Figure 2). Similarly, the response of the second group of Dox‐resistant HeLa cells and parental HeLa cells incubated with Dox contained fibrous mat (F + D) was not significantly different from the viability of the first group of cells. The viability of parental HeLa and Dox‐resistant HeLa cells reduced significantly (at *P* ≤ .05) to 30% and 50%, respectively, in the third group when incubated with fibrous mat (without Dox) and AMF generated heat (F + H). Further, the response of HeLa cells and Dox‐resistant HeLa cells was not similar in the fourth group when cells incubated with Dox contained fibrous mat and AMF (F + H + D). The viability of parental HeLa cells reduced to 15% when exposed to heat and Dox simultaneously as compared to the viability of Dox‐resistant HeLa cells (~50%).

### 3.3 In vivo hyperthermia treatment

**FIGURE 3:**
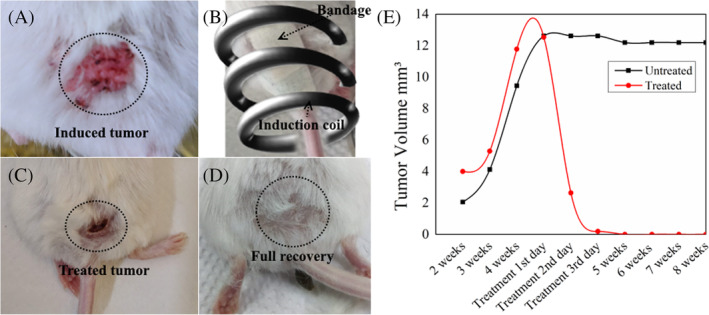
The photographic images of the tumor: (A) Before treatment. (B) During treatment: The tumor is covered with a bandage and placed under the induction coils. (C) After five doses of hyperthermic cycles for 15 minutes. (D) After 2 weeks of the treatment. (E) The change in volume of the tumor over time with or without treatment

The efficacy of the magnetic fibrous mat‐based bandages was tested for the treatment of skin cancer on the BALB/c mice using two‐stage chemical carcinogenesis initiated by DMBA and promoted by PMA. Figure 3A shows the formation of the skin tumor after 5 to 6 weeks of chemical carcinogenesis. The nanofibrous bandage was placed on the tumor of TB group of mice and subjected to an external AMF (Figure 3B) for few minutes to achieve 45°C around the bandage and maintained this temperature for 15 minutes. In order to avoid the possibility of recurrence of cancer, the mice were given up to five heating cycles for 15 minutes (first 3 days, and then on fifth and seventh day). As shown in Figure 3C, there were no surroundings burning to the healthy skin near the tumor area and tumor completely disappeared with a small wound. The mice were healed fully after around 3 weeks of the treatment without further aids. As shown in Figure 3D, there is no sign of the inflammation, and hair growth was also restored in the treated area. The tumor volume in the case of untreated mice remained unchanged after suspending the application of the promoter TPA (Figure 3E).

**FIGURE 4:**
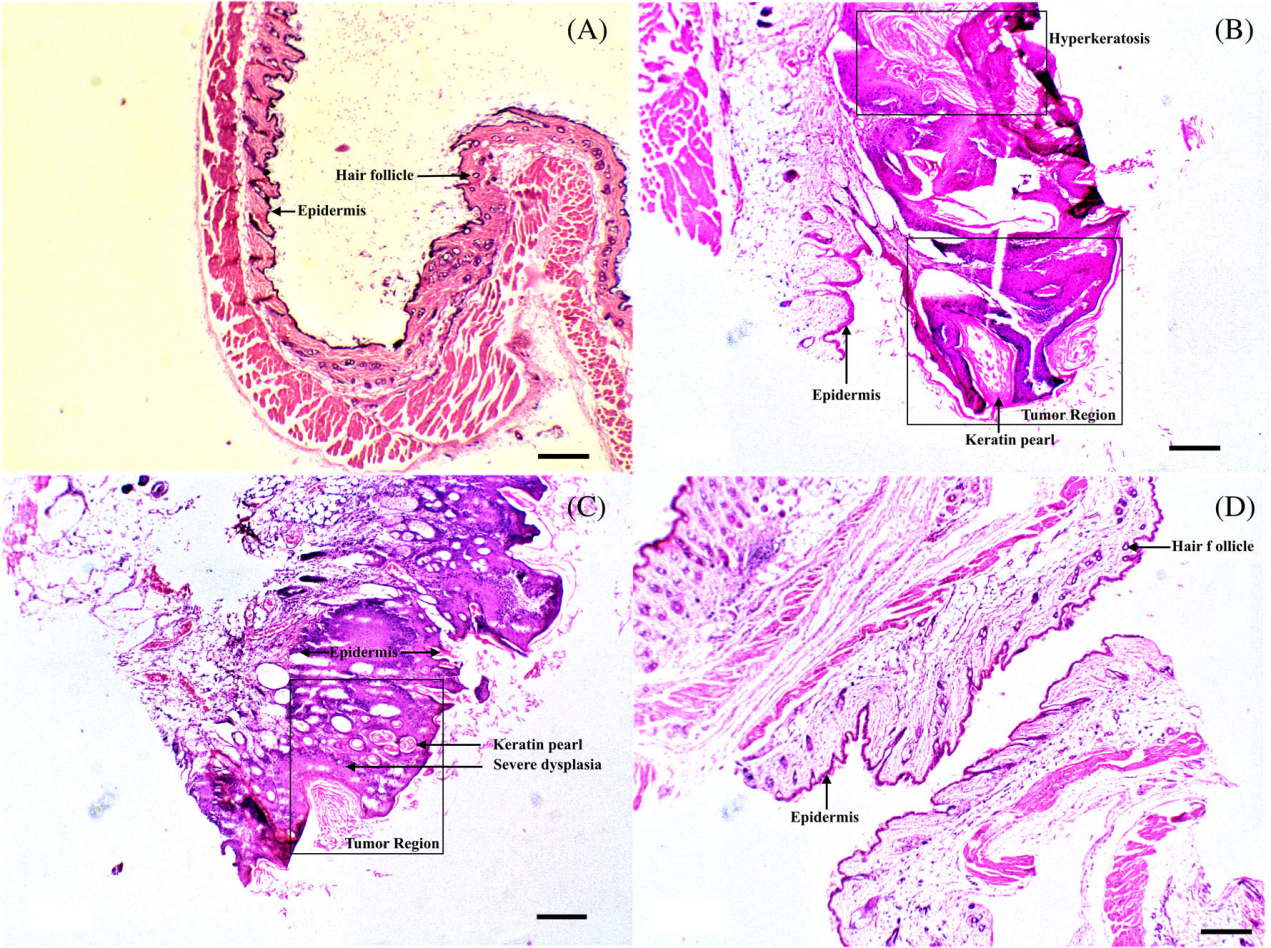
Represented H&E images of (A) C, control group. (B) UT, untreated mice group. (C) TH, treated without bandage only with AMF. (D) TB, treated mice group with bandage and AMF. Scale bar 500 μm. AMF, alternating magnetic field; H&E, hematoxylin and eosin

In order to investigate the effect of heat on the underneath layers of skin, after 30 days of the treatment, the mice were sacrificed, and the tumor regions in treated and untreated mice were fixed in formalin and embedded in paraffin for histochemical analysis (Figure 4). The tumor region of the untreated mice in the UT group (Figure 4B) and mice exposed to AMF without bandage in the TH group (Figure 4C) exhibited (a) severe dysplasia, that is, the abnormal cells grow in the full thickness of the epithelium,^39^ (b) presence of keratin pearls, (c) hyperkeratosis, (d) thickening and inflammation in epidermis, and (e) loss of hair follicles in the dermal region when compared with the tumor region in treated mice with bandages in the TB group (Figure 4D). After treatment, there is no thickening, inflammation in the epidermis, and shows the presence of hair follicles, which resembles much closer to the control mice sample. The kidney and the liver of all the mice were fine after treatment as there is no significant difference in the creatinine, urea, serum glutamic oxaloacetic transaminase (SGPT), serum glutamic pyruvic transaminase (SGOT), bilirubin, and alkaline phosphatase levels when compared to the control group (see Table 1).

**TABLE 1:** The blood test report for liver and kidney function

Groups	Creatinine	Urea	SGPT	SGOT	Total‐Bilirubin	Direct‐Bilirubin	Alkaline Phosphatase
Control (C)	0.65 ± 0.05	37.80 ± 18.92	42.60 ± 12.01	50.40 ± 15.78	22.40 ± 15.30	18.38 ± 15.34	152.80 ± 20.29
Untreated (UT)	0.72 ± 0.07	41.25 ± 20.12	49.00 ± 19.70	56.25 ± 20.48	26.25 ± 6.65	22.00 ± 9.27	148.50 ± 11.38
Treated (TH without bandage)	1.00 ± 0.28	35.00 ± 8.48	30.50 ± 2.12	36.00 ± 00	23.50 ± 2.12	15.50 ± 0.70	162.00 ± 8.48
Treated (TB)	0.83 ± 0.19	36.37 ± 11.81	46.00 ± 20.36	54.50 ± 21.03	21.33 ± 8.04	15.48 ± 8.62	173.25 ± 30.88
After full recovery	0.95 ± 0.16	35.00 ± 5.9	32.75 ± 213.45	38.12 ± 28.92	20.87 ± 6.77	16.12 ± 6.13	172.63 ± 24.07

Abbreviations: SGOT, serum glutamic oxaloacetic transaminase; SGPT, serum glutamic pyruvic transaminase.

## 4 DISCUSSION

Hyperthermia is an effective technique to treat cancer, yet the delivery of localized heat energy uniformly to kill cancerous cells is difficult to achieve. In the present study, the PCL‐Fe_3_O_4_ fibrous mat‐based bandages are fabricated for non‐invasive skin cancer treatment. The electrospun fibers are continuous with 100 to 1000 nm diameter range, and the Fe_3_O_4_ NPs are firmly embedded in the PCL matrix (Figure 1A and B). The fibrous mat dissipated heat energy and increased surrounding temperature when subjected to AMF (Figure 1C). As shown in Figure 1E, the mechanism of heat loss from the spherical Fe_3_O_4_ NPs in the presence of AMF is predominantly due to the magnetic hysteresis losses caused by the alignment of magnetic dipoles parallel to the external magnetic field.^30^, ^37^ The reversal of dipole moments direction in an AMF at higher frequencies loses energy equivalent to the area under the hysteresis curve. This loss of energy in the form of heat uses in the hyperthermia therapy. It is evident from Figure 2 that the HeLa cells can be killed effectively by hyperthermia using PCL‐Fe_3_O_4_ fibers with the application of AMF. The percentage of both Dox‐resistant and parental HeLa dead cells was higher (>50%) after the exposure to AMF compared to cells without AMF exposure. When cells are incubated with fibrous mat in the presence of AMF, the continuous flow of heat by fibrous mat to the culture media and cells, increases surrounding temperature. The elevated temperature damages the cellular fluidity, deoxyribonucleic acid (DNA), cytoskeleton organization, and membrane, which leads to apoptotic/necrotic cell death in vitro.^40^

Hu et al reported that the low dosage of hyperthermia (LDH) could reverse the chemo‐resistant behavior of SCC cell line SCCVII after 30 to 60 minutes exposure of heat.^22^ They found that the LDH promoted a 25.22‐fold increase of p53 mRNA, an 18.08‐fold increase of Bax mRNA, and 28.7% decreased MDR1 expression in vitro, leading to an increase in the apoptotic cell population. Moreover, it is known that the required Dox concentration to kill cancerous cells is 10 μg/mL. Thus, the 20 μg of Dox in the 10 mg of the fibrous mat was incorporated during electrospinning in order to maintain the minimum Dox concentration during incubation with cells.^41^, ^42^, ^43^ We found previously, the release of Dox from the fibers was 1.8‐fold higher in the presence of AMF as compared to without AMF exposure.^37^ The encapsulated Dox in the fibers released through diffusion, and the rate of diffusion increases at the elevated temperature, consequently a higher amount of Dox is released from the fibers in the presence of AMF. Despite the required Dox concentration (10 μg/mL) in the culture media, there was no significant parental and Dox‐resistant HeLa cell death observed (Figure 2) in the absence of AMF due to the less incubation (10 minutes) of cells with Dox.^37^ Previously, we found that the percent of dead HeLa cells increased when cells were incubated with Dox contained fibers for more than 3 days, as the chemo drug takes time to enter inside, cleave DNA, and kill the cell.^37^ Likewise, there was no significant reduction in the percent viability of Dox‐resistant HeLa cells on the application of heat and drugs (F + D + H) simultaneously when compared to cells only exposed to heat (F + H). On the other hand, the reduction in the viability in the case of parental HeLa cells was significantly higher when cells were incubated with both drug and heat simultaneously (F + D + H). This disparity in the cell response could be again due to the lack of enough incubation time of cells after the exposure of heat. The reversal of cellular resistance to drugs takes place after 24 hours of heat exposure; therefore, the viability of cells immediately after incubation with heat and Dox for 10 minutes did not alter the percentage of dead cells in the case of Dox‐resistant cells.^22^ However, the enhanced Dox activity at a higher temperature led to more cell death in the case of parental HeLa cells.

**FIGURE 5:**
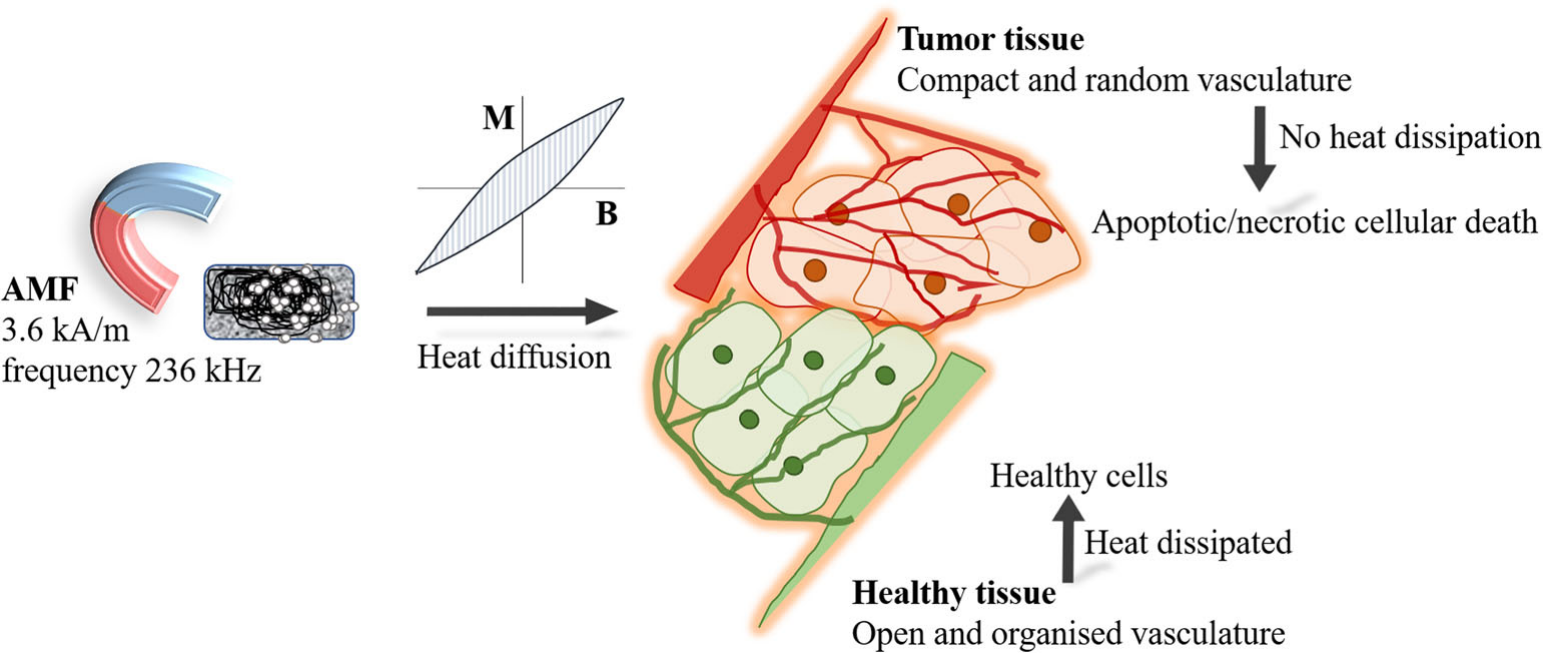
Schematic representation of mechanism of heating and cellular death at elevated temperature in the presence of AMF. AMF, alternating magnetic field

The efficacy of bandages to treat skin cancer was tested in vivo using BALB/c mouse model. The skin tumors were induced on the mice's skin by two chemicals: DMBA and PMA. The induced tumors (eg, Figure 3A) were treated with hyperthermia at 45°C for 15 minutes using magnetic fibrous bandages (Figure 1C). As shown in Figure 3B, the induced tumor near the tail of the mouse (TB group) was placed inside the induction coil after placing a bandage on the tumor. The frequency in the interval 50 kHz < *f* < 1 MHz and amplitude of the applied field (*H* < 15 kA/m) are recommended for the safety and comfort of the patients during treatment.^44^ Furthermore, the number of heating cycles is also a critical parameter to determine the recurrence of tumors and formation of scar after treatment. The fewer heating cycles with low temperature can induce early cellular apoptosis (non‐lethal damage) which can result in recurrence of cancer.^45^ Moreover, intense AMF (>500 kHz and 15 kA/m) with one or two heating cycles can lead to severe burns to the tissue and forms a scar. Therefore, all the mice in the TB group were treated with up to five heating cycles for 15 minutes on first, second, third, fifth, and seventh day with an optimal and human tolerance limit of AMF, that is, 3.6 kA/m and frequency 236 kHz. After the treatment, the size of the tumor reduced significantly (Figure 3C), and all the mice recovered completely after 2 weeks of the treatment (Figure 3D). There was no reduction in the size of the tumor when mice were exposed to AMF without bandage (TH group), which confirms that the generation of heat through bandages killed the cells instead of external AMF. Additionally, all the mice showed a similar pattern of decrement in the tumor volume during treatment, while in other cases, the volume of the tumor did not change over time (Figure 3E). As mentioned above, the generation of heat is dependent on the magnetic materials properties and external AMF strength. However, unlike the protein denature and DNA damage by direct heat flow to cells in vitro, the diffusion of heat in vivo depends on various other parameters, such as, the temperature of heat source, tissue thermal conductivity, tissue specific heat, and blood tissue density.^46^ As presented in Figure 5, the vasculature around the tumor is very compact and random with acidic microenvironment to provide the nutrients for the growth of fast‐growing cells as compared to nearby healthy cells. The elevated surrounding temperature increases the blood flow and ruptures the compact vasculatures of the tumor tissue, which hinders the nutrient supplies to growing cells and results in cell death.^40^ On the contrary, the healthy tissue with organized vasculature can maintain its temperature by the dissipation of heat, and there was no irreversible damage to the healthy cells were noted. The H&E stain analysis further confirmed the absence of any abnormal cell growth at the site of treatment in any of the mice contrary to untreated mice (UT group and TH group) where the abnormal cells were grown in the full thickness of the epithelium and formed keratin pearls and lost hair follicles (Figure 4B and C.

Hou et al studied the efficacy of magnetic hydroxyapatite nanoparticles (mHAP) for the treatment of cancer with hyperthermia using a mouse model.^47^ The mHAP NPs were injected at the tumor site, followed by the application of the AC magnetic field. They observed that the tumor shrank rapidly during treatment, and the animal recovered after 2 weeks of the treatment. However, they also observed elevated AST and ALP levels, which indicated a damaged liver because of the metabolism of HAP/mHAP. In the present case, the embedded Fe_3_O_4_ NPs superficially transfer the heat to cells without internalization. Thus, no such side effects observed due to the metabolism of MNPS. The blood test results further ruled out any adverse effect of the therapy. The blood test shows similar values for all the cases, which indicated that the tumor was superficial, and the hyperthermia did not affect any of the organs in the animal. There was no sign of recurrence of the tumor and inflammation seen in any group mice after 2 weeks of the treatment. In hyperthermia therapy, the uniform dissipation and penetration of heat at the site of tumor play a significant role in achieving full recovery. The uniform distribution of MNPs in the PCL fibrous matrix results in even heat generation and dissipation. The candidacy of designed bandage for skin cancer treatment is still in the beginning stage, and other supportive studies need to be done before it could use for any clinical application. Furthermore, the current study shows that the use of bandage for superficial tumors; however, with some modifications and minimal surgery, it could be used for the treatment of other types of cancers as well as in the future.

## 5 CONCLUSIONS

The localized heating to treat skin cancer using a magnetic material is a compelling approach. We have shown that the magnetic fibrous mat‐based bandage can effectively treat skin cancer with the help of an external AC magnetic field. As embedded Fe_3_O_4_ NPs superficially interact with cells without internalization, no long term toxicity due to MNPs was observed. The in vitro study confirms the hyperthermia could also be an effective technique to kill Dox‐resistant cells. Furthermore, the skin tumors on the BALB/c mice were treated using fabricated bandages with five heating doses combined with optimal AMF strength (3.6 kA/m and frequency 236 kHz) to maintain localized temperature 45°C for 15 minutes. With these doses, the irreversible necrotic tumor cell death was achieved with no sign of recurrence of cancer and inflammation or burns to the healthy tissue post‐therapy. The complete recovery of the treated mice tumor was achieved within 2 weeks of the treatment with the designed bandages combined with specific parameters. The present study confirms the efficacy of the bandages with a specific parameters, however, there is still a possibility of the other effective treatment doses that can fully treat cancer.

## AUTHOR CONTRIBUTIONS

**Kaushik Suneet:** Data curation; formal analysis; investigation; methodology; writing‐review and editing. **Tamasa De:** Investigation; methodology; validation; writing‐review and editing. **Annapoorni Rangarajan:** Conceptualization; funding acquisition; methodology; writing‐review and editing. **Shilpee Jain:** Conceptualization; data curation; formal analysis; funding acquisition; methodology; project administration; resources; supervision; writing‐original draft; writing‐review and editing.

## CONFLICT OF INTEREST

The authors have stated explicitly that there are no conflicts of interest in connection with this article.

## ETHICAL APPROVAL

Animal protocols were approved by the Institutional Animal Ethics Committee (IAEC) and animals were maintained according to Committee for the Purpose of Control and Supervision of Experiments on Animals (CPCSEA) guidelines (a light/dark cycle of 12 hours, temperature 22°C ± 2°C).

## Data Availability

Data are available on request from the authors.
